# Two different presentations of de novo variants of *CSNK2B*: two case reports

**DOI:** 10.1186/s13256-021-03184-8

**Published:** 2022-01-05

**Authors:** Matheus V. M. B Wilke, Bibiana M. Oliveira, Alessandra Pereira, Maria Juliana R. Doriqui, Fernando Kok, Carolina F. M. Souza

**Affiliations:** 1 Medical Genetics Service, Hospital de Clínicas de Porto Alegre, Rua Ramiro Barcelos, 2350 – 3º andar, Porto Alegre, RS 90035-007 Brazil; 2 Post Graduate Program in Medical Sciences, Universidade Federal do Rio Grande do Sul, Porto Alegre, RS Brazil; 3 Post Graduate Program in Genetics and Molecular Biology, Universidade Federal do Rio Grande do Sul, Porto Alegre, RS Brazil; 4 Mendelics Genomic Analysis, São Paulo, SP Brazil; 5 Pediatrics Service, Neuropediatrics, Hospital Moinhos de Vento, Porto Alegre, RS Brazil; 6 Hospital Infantil Dr. Juvêncio Mattos, São Luís, MA Brazil

**Keywords:** Epilepsy, Hypotonia, Dysmorphic features, Case report

## Abstract

**Background:**

Poirier–Bienvenu neurodevelopmental syndrome is a neurologic disorder caused by mutations in the *CSNK2B* gene. It is mostly characterized by early-onset seizures, hypotonia, and mild dysmorphic features. Craniodigital syndrome is a recently described disorder also related to *CSNK2B*, with a single report in the literature.

**Objective:**

To report two unrelated cases of children harboring *CSNK2B* variants (NM_001320.6) who presented with distinct diseases.

**Case report:**

Case 1 is a 7-month-old, Caucasian, female patient with chief complaints of severe hypotonia and drug-refractory myoclonic epilepsy, with a likely pathogenic de novo variant c.494A>G (p.His165Arg). Case 2 is a 5-year-old male, Latino patient with craniodigital intellectual disability syndrome subjacent to a de novo, likely pathogenic variant c.94G>T (p.Asp32Tyr). His dysmorphic features included facial dysmorphisms, supernumerary nipples, and left-hand postaxial polydactyly.

**Conclusion:**

This report suggest that the *CSNK2B* gene may be involved in the physiopathology of neurodevelopmental disorders and variable dysmorphic features.

## Introduction

Poirier–Bienvenu neurodevelopmental syndrome (POBINDS; OMIM #618732) is a recently described disorder characterized by hypotonia, seizures, and developmental delay [1]. POBINDS is caused by mutations in the *CSNK2B* gene (located at 6p21.33), which encodes the beta subunit (CK2β) of the casein kinase 2 enzyme (CK2). It has been reported that the *CSNK2B* gene is neither susceptible to missense mutations (*Z* = 3.83) nor loss of function (pLi 0.92; observed/expected = 0.08; 95% confidence interval 0.03–0.38). In fact, all cases reported have been subjacent to de novo mutations and caused variants that lead to loss of function [2].

Dysmorphic features other than those originally reported have recently been associated with craniodigital syndrome (CDS), a condition that can be distinguished from POBINDS [3]. However, these dysmorphic features are not fully characterized [4].

Th work aims to detail the clinical manifestations of patients with two *CSNK2B* variants presenting with distinct phenotypes, one with the phenotype closer to POBINDS and another with the phenotype closer to CDS. To the best of our knowledge, only two cases have described patients with similar dysmorphic features. We also discuss possible pathogenic mechanisms based on a literature review, emphasizing the necessity to better elucidate the clinical phenotype spectrum related to the *CSNK2B* gene.

## Case report

### Case #1: Hypotonia and neonatal myoclonic spasm

A 7-month-old, female Caucasian patient presented with neurodevelopmental delay followed by epilepsy with unaltered electroencephalogram (EEG). The patient is the second child of a nonconsanguineous couple, having an 11-year-old healthy brother. No other family member had a medical history of neurological or genetic disorders. Although the pregnancy was uneventful, the patient was delivered by cesarean due to placental detachment with a gestational age of 39 + 1 weeks. The patient was a full-term infant with birth weight of 3.4 kg and APGAR score of 6 and 8 in the first and fifth minutes, respectively. Due to myoclonic movements, hypotonia, and cyanosis, the patient was admitted to the neonatal intensive care unit.

**Fig. 1:**
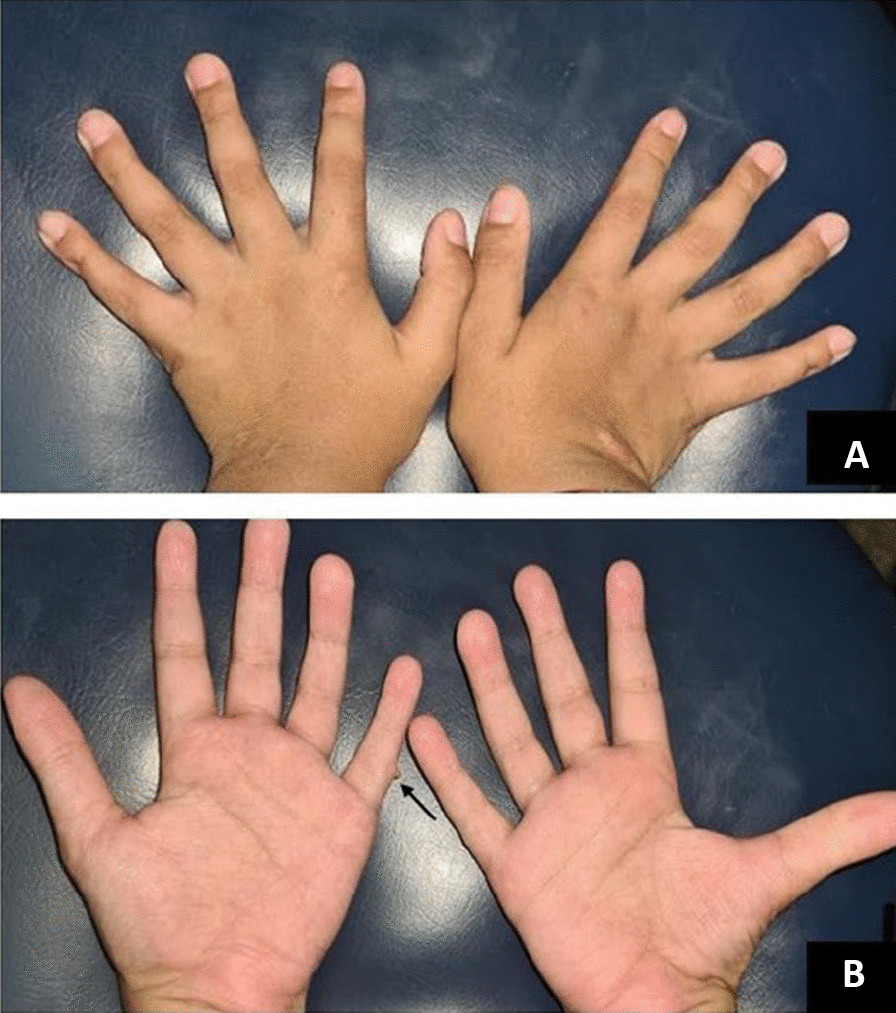
Hands of patient #2: The patient from the second case report showed fifth short fingers, tapered distal phalanges of fingers, left hand postaxial polydactyly (surgically corrected, pointed by the arrow) and nail hypoplasia in the (**A**) and (**B**) panels

At the age of 23 days, the patient was referred to a Reference Center in Rare Genetic Diseases at the Porto Alegre University Hospital. The epileptiform crisis evolved to recurring focal motor seizures in the upper limbs with simultaneous apnea. The physical evaluation revealed marked global hypotonia and dysmorphic features such as midfacial hypoplasia, bilateral strabismus, tongue protrusion, and dysplastic ears (overfolded helix) as shown in Fig. 1.

The patient underwent a metabolic genetic investigation, which included normal urinary organic acids and quantitative plasma amino acids. Empiric treatment with biotin and pyridoxine failed to result in any improvement of her clinical picture. Neuroimaging study (magnetic resonance imaging, MRI) did not reveal any abnormality and the EEG was unaltered even when it was performed during the myoclonic movements. Due to global hypotonia, further investigation ruled out Prader Willi Syndrome, Pompe disease, and congenital disorders of glycosylation. Karyotype was also unchanged, and the chromosomal microarray revealed a duplication of the 1p31.3 (61236531_61661276), which was considered a variant of uncertain significance.

Laryngeal fiberscopic evaluation revealed an obstructive laryngomalacia. The patient underwent a supraglottoplasty at the age of 1 month. Despite the procedure, the spasms persisted. The patient was then discharged from the hospital for further follow-up in the outpatient clinic with the diagnostic hypothesis of sleep myoclonus.

At the age of 7 months, physical examination revealed severe global development delay (inability to lift the head) due to truncal hypotonia. Her epilepsy had worsened (intensity and duration of the episodes) being considered a pharmacoresistent myoclonic epilepsy triggered by both auditory and visual stimuli.

The exome sequencing revealed a de novo likely pathogenic *CSNK2B* variant (NM_001320.6). The variant c.494A>G (p.His165Arg) is present in exon 6 out of 7 exons, in a highly conserved amino acid and it is not observed in The Genome Aggregation Database (gnomAD). In silico tools predict the variant to be deleterious (SIFT: Damaging 0; Revel: Deleterious low; CADD Score: 26.1). The variant is found in the Human Gene Mutation Database (HGMD) as a disease-causing mutation (DM). No other pathogenic variation was found in other epilepsy candidate genes.

### Case #2: Hypotonia and dysmorphisms

A 5-year-old, male Latin patient was referred for genetic evaluation at the age of 13 months, with the chief complaints of dysmorphisms, hypotonia, and developmental delay.

The patient was born from a nonconsanguineous young couple. He was also delivered by cesarean section due to placental detachment, with a gestational age of 41 weeks. He was a full-term infant with birth weight of 3.4 kg and APGAR score of 9 in the first and fifth minutes.

The patient’s motor development was considered delayed: the patient sustained his head at the age of 6 months, sat unassisted at the age of 8 months, and walked independently at 2.4 years. At 9 months of age, the patient presented with acute episodes of muscle tone loss associated with abnormal eye movements. The EEG performed at 1 year showed disorganized basal activity, which was considered to be related to brain immaturity. The sleep-related graph elements did not show any epileptiform paroxysmal waves.

Physical examination revealed axial hypotonia, microbrachycephaly, low nasal bridge, anteverted nares, malar hypoplasia, high and narrow palate, duplicated upper right incisor, prominent and dysplastic ears, and supernumerary nipples. Examination of the extremities showed short fifth fingers, tapered distal phalanges of fingers, left hand postaxial polydactyly (surgically corrected), and nail hypoplasia, as shown in Fig. 1.

Genetic evaluation included karyotype and chromosomal microarrays, which were unaltered. Exome sequencing identified a de novo missense pathogenic *CSNK2B* variant (NM_001320.6) in heterozygosis. The variant c.94G>T is present in exon 3 out of 7 exons, with a highly conserved amino acid sequence, which are not described in the gnomAD. In silico tools predicted that the variant is likely deleterious (SIFT: Damaging 0; Revel: Deleterious high; CADD Score: 32). In HGMD, the variant position was already reported, leading to a different amino acid substitution (c.94G>A; p.Asp32Asn) and subjacent to the same phenotype. Sanger sequencing confirmed the presence of the variant in the patient but not in the parents.

## Discussion

After the initial report of POBINDS [1], two subsequent papers documented the medical history of three unrelated patients with marked impaired global development and complex partial seizures before 2 months of age [5, 6]. The refractory seizures observed in these patients included focal evolving to generalized tonic–clonic episodes, and epileptic encephalopathy. Epilepsy in POBINDS is also characterized by unaltered EEG and MRI. Being drug resistant, tonic–clonic seizures are the most frequent presentation [2, 7, 8]. The absence of a specific imaging pattern corroborated in both of our cases to a delayed diagnosis, as exemplified in Case #1 where sleep myoclonus was one of the differential diagnosis owing to the unaltered EEG.

Few *in vivo* studies have demonstrated the interaction of CK2 and voltage channels that could explain the epilepsy seen in these patients. Potassium channels are the most diverse group of the ion channel family. These channels are not only involved in shaping the action potential, but also in neuronal excitability and plasticity. One study using transfected Chinese hamster ovary cells expressing Kv3.1 (a potassium channel with a very high threshold) demonstrated the importance of its phosphorylation by CK2. When CK2 was inhibited, there was a decrease in the phosphorylation of the Kv3.1 channels, leading to the decline in its electric potentials [9, 10]. Although speculative, it might be related to the etiology of seizures found in these patients.

The most striking clinical feature of Case #2 was the dysmorphic features that were recently associated with CDS, which could be distinguished from POBINDS [3]. A recently published paper reported the case of a young male Japanese patient with a deletion of 6p21.33 (which encompasses the *CSNK2B* gene). The patient displayed very similar features to patient #2, including large low-set ears, downslanted palpebral fissures, flared eyebrows, wide-base nose, and flat philtrum with thin upper lip [11] (for further details, see [8] and Table 1). Collectively, these cases corroborate the loss of function as plausible mechanisms described for both POBINDS and CDS.

**Table 1:** Comparison of the clinical phenotype of patients described in this paper with those in [11]

Clinical Features	Case 1	Case 2	[11]
Genotype	c.494A>G (p.His165Arg)/–	c.94G>T(p.Asp32Tyr)/–	6p.21.33 deletion/–
Perinatal history	Placental detachment	Placental detachment	Uneventful
Hypotonia	Yes	Yes	Yes
ID/DD	Yes	Yes	Yes
Gross motor development delay	Yes	Yes	Yes
Development of speech and language	Yes	No	Yes
Facial dysmorphisms	Midfacial hypoplasia, bilateral strabismus, lingual protrusion, and over-folded helices	Microbrachycephaly, low nasal root, anteverted nostrils, malar hypoplasia, high and narrow palate, duplicated upper right incisor, prominent and dysplastic ears	Relative macrocephaly. Large low-set ears, downslanting palpebral fissures, flared eyebrows, wide-base nose, and flat philtrum with thin upper lip
Other dysmorphisms	No	Supernumerary nipples, short fifth fingers, distal tapering of fingers, left hand postaxial polydactyly, and nail hypoplasia	ND
Age at seizure onset	Newborn	9 months	ND
Seizure types	Myoclonic spasms	Atonic Seizures	ND
Familiar recurrence	No	No	No
EEG	Normal	Disorganized base activity	ND
Brain image	Normal	Normal	Incomplete hippocampal folding
Other	Obstructive laryngomalacia	No	Asthma attacks and food allergies

CK2 is a heterotrimeric enzyme consisting of two catalytic CK2α or CK2α′ subunits and two regulatory β subunits. It also contains several distinct domains. The three main domains are Asp/Glu-enriched (acidic), zinc-binding (where the dimerization of β subunit occurs), and α-subunit-interaction domains [12, 13]. Even though it is considered a regulatory subunit, CK2β is highly conserved. Previous studies have demonstrated that if this regulatory subunit is excessively synthesized, there is a trend to the formation of of CK2β dimers, which are, in turn, a prerequisite for the formation and correct function of the whole enzyme [12, 14].

**Fig. 2:**
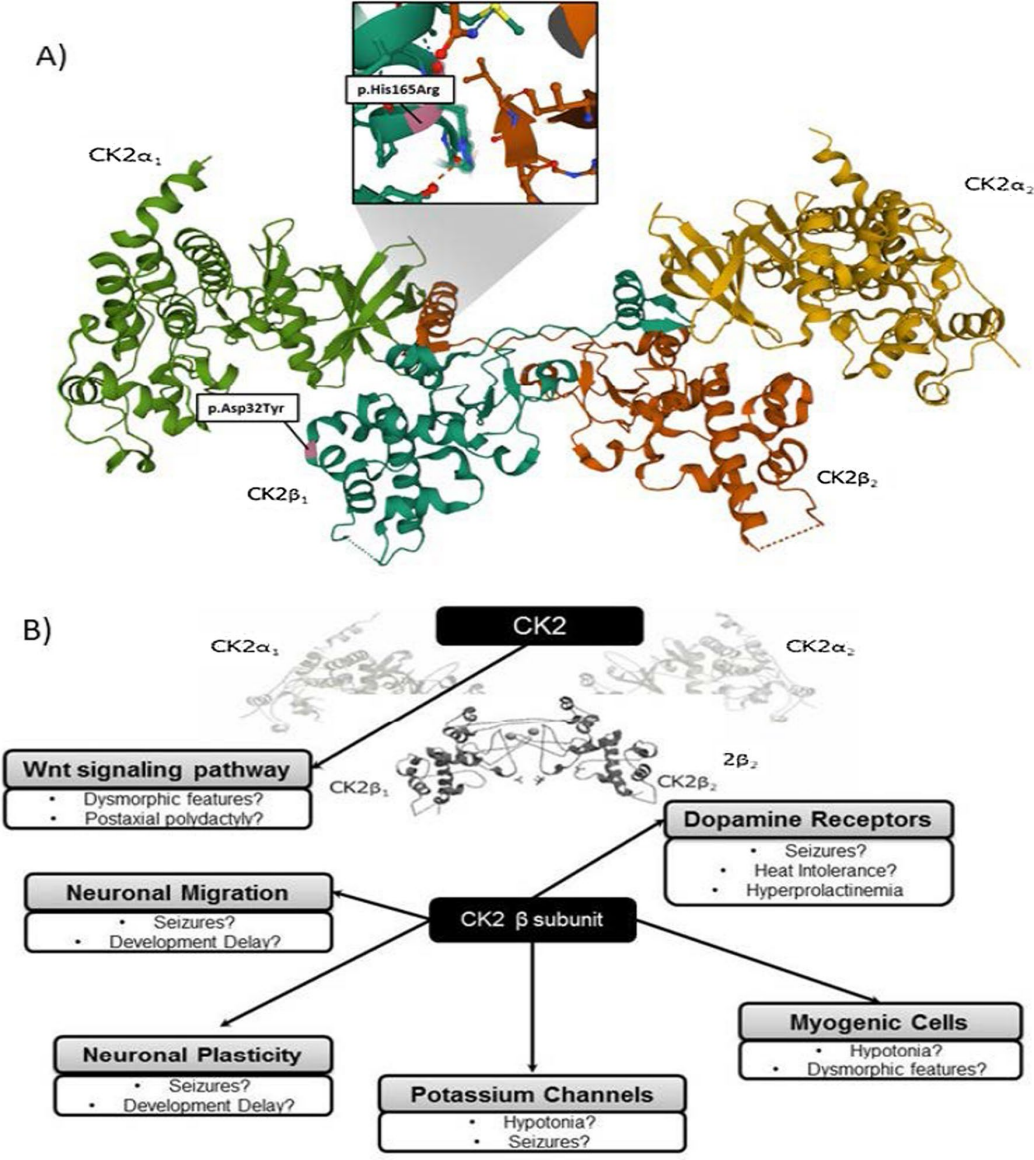
Hypothetical association among the patients’ phenotypes to the *CSK2B* variants. In **A**, the Ribbon diagram illustrates the high-resolution structure of tetrameric CK2 and the location of the variants. p.His165Arg is located in close interaction between CK2β1 and CK2β2 and p.Asp32Tyr to the alfa1 catalytic subunit. In the crystal structure of full-length symmetric CK2 (α2β2) holoenzyme (PDB ID: 4MD7) (**B**), the β subunit interacts with different systems. Gray boxes contain previously described roles of CK2 and white boxes detail the putative association with the clinical manifestation of the reported patients.

It is believed that the zinc-binding domain might be a hotspot for pathogenic variants and that the α-subunit-interaction domain might be the one related to refractory myoclonic epilepsy [2, 15]. The closest zinc binding site in the variant-related protein from patient#1 is a cysteine in position 137. However, p.His165Arg, according to crystal structure, is in close interaction with the complementary beta subunit, as seen in Fig. 2. The dimers of CK2β are located at the core of the tetrameric CK2 complexes and we hypothesize that symptoms observed in patient #1 might be due to alterations in this beta-beta interaction, subsequently leading to a disruption in the CK2β dimerization.

Two de novo missense mutations (p.Asp32Asn and p.Asp32His) in the same *CSNK2B* codon, which is mutated in patient #2, have recently been associated with CDS [3, 7]. Due to the modification of the CK2 structure, the closest CK2 catalytic subunit would be position 32, which could exert a more severe dysmorphic phenotype. One study using whole transcriptome and whole phosphoproteome profiling demonstrated that variants in this protein amino acid position caused an up-regulation of *CSNK2B* gene expression at transcript and protein levels, resulting in impaired cross talk between α and β subunits of CK2 [3]. Both hypotheses, however, must be further demonstrated by functional studies. A definite association between phenotype and genotype in *CSNK2B* gene-related epilepsy has still not been described [2].

A hypothesis for the POBINDS-related “brain-centered symptoms” could be raised after preclinical research on the CK2 function in animal cell models. The CK2 subunit appears to be constitutively active, being a target for dopamine D1 receptors signaling pathway [1, 16]. The resulting alterations in the dopaminergic system (as per increased D1 receptor signaling and/or dopamine production) has also been found in patients with juvenile myoclonic epilepsy [8], possibly contributing to understanding of myoclonic epilepsy, as well as the excessive sweating observed in the patient in case #1.

Additionally, a study using GN11 cells as a model of immature migrating neurons, demonstrated that CK2β was involved in cell migration and microfilament/microtubule organization in neurons, thus playing a role in neurodevelopmental steps such as neural differentiation, neuritogenesis, and synaptic plasticity [15, 17]. In line with these findings, studies using β subunit CK2 knockout cell lines (KOβ) pointed out that the β subunit may serve as an anchorage point for other kinases, being involved in the myogenic commitment of C2C12 (myogenic cell lines in murines) by regulating MyoD expression independently from the catalytic subunits [15, 17]. In a C2C12 model, CK2β contributes to a variety of cell cycle processes such as proliferation and cellular transport *in vitro*. Collectively, these findings might contribute to the elucidation of the hypotonia presented by both patients. CK2 has also been implicated in organogenesis, playing a role in heart embryogenesis and limb bud differentiation via the Wnt signaling pathway. Further studies are warranted to determine the association of this role to the limb dysmorphisms found in the patient from the second case [4].

## Conclusion

To date, 16 patients have been reported in the literature with POBINDS and 2 patients have been reported with craniodigital intellectual disability syndrome related to mutations in the *CSNK2B* gene. The present cases may assist in the diagnosis of POBINS when patients present with refractory epilepsy, hypotonia, and dysmorphic features. Further investigation is warranted to better understand the interaction of CK2 and the neuronal and neuromuscular systems. We suggest that screening of *CSNK2B* could be included in the most common gene panels for epilepsy.

## Data Availability

Data was retrieved from patients medical files from Hospital de Clinicas de Porto Alegre (Porto Alegre – RS, Brazil), Hospital Moinhos de Vento (Porto Alegre – RS, Brazil), Laboratorio Mendelics (Sao Paulo- SP Brazil) with consent.

## Abbreviations

ACMG: American College of Medical Genetics
CDS: Craniodigital syndrome
CMA: Chromosomal microarray
CK2: Casein kinase 2 enzyme
CK2β: Casein kinase 2 enzyme beta subunit.
EEG: Electroencephalogram
gnomAD: The Genome Aggregation Database
HGMD: Human Gene Mutation Database
LOF: Loss of function
POBINDS: Poirier–Bienvenu neurodevelopmental syndrome
